# Arm rotated medially with supination – the ARMS variant: description of its surgical correction

**DOI:** 10.1186/1471-2474-10-32

**Published:** 2009-03-16

**Authors:** Rahul K Nath, Chandra Somasundaram, Sonya E Melcher, Meera Bala, Melissa J Wentz

**Affiliations:** 1 Texas Nerve and Paralysis Institute, Houston, TX, USA

## Abstract

**Background:**

Patients who have suffered obstetric brachial plexus injury (OBPI) have a high incidence of musculoskeletal complications stemming from the initial nerve injury. The presence of muscle imbalances and contractures leads to typical bony changes affecting the shoulder, including the SHEAR (Scapular Hypoplasia, Elevation and Rotation) deformity. The SHEAR deformity commonly occurs in conjunction with Medial Rotation Contracture (MRC) of the arm. OBPI also causes muscle imbalances at the level of the forearm, that lead to a fixed supination deformity (SD) in a small number of patients. Both MRC and SD will cause severe functional limitations without surgical intervention.

**Methods:**

Fourteen OBPI patients were diagnosed with MRC of the shoulder and SD of the forearm along with SHEAR deformity during a 16 month study period, with eight patients available to long-term follow-up (age range 2.2 – 18 years). Surgical correction of the MRC was performed as a triangle tilt or humeral osteotomy depending on the age of the child, after which, the patients were treated with a radial osteotomy to correct the fixed supination deformity. Function was assessed using the modified Mallet scale, examination of apparent supination and appearance of the extremity at rest.

**Results:**

Significant functional improvements were observed in patients with surgical reconstruction. Mallet score increased by an average of 5.2 (p < 0.05). Overall forearm position was not significantly changed from an average of 5° to an average of 34° maximum apparent supination after both shoulder rotation and forearm rotation corrective surgeries.

**Conclusion:**

The simultaneous presence of two opposing deformities in the same limb will visually offset each other at the level of the wrist and hand, giving the false impression of neutral positioning of the limb. In reality, the neutral-appearing position of the hand indicates a fixed supination posture of the forearm in the face of a medial rotation contracture of the shoulder. Both of these deformities require surgical attention, and the presence of concurrent MRC and SD should be monitored for in OBPI patients.

## Background

Secondary deformities are common following obstetric brachial plexus injury (OBPI). Two well-described secondary deformities are the medial rotation contracture (MRC) of the arm and the fixed supination deformity (SD) of the forearm. Each has been described individually, but the simultaneous presence of both in the same patient, which we term Arm Rotated Medially with Supination, or the ARMS variant of MRC, has not previously been emphasized.

**Figure 1:**
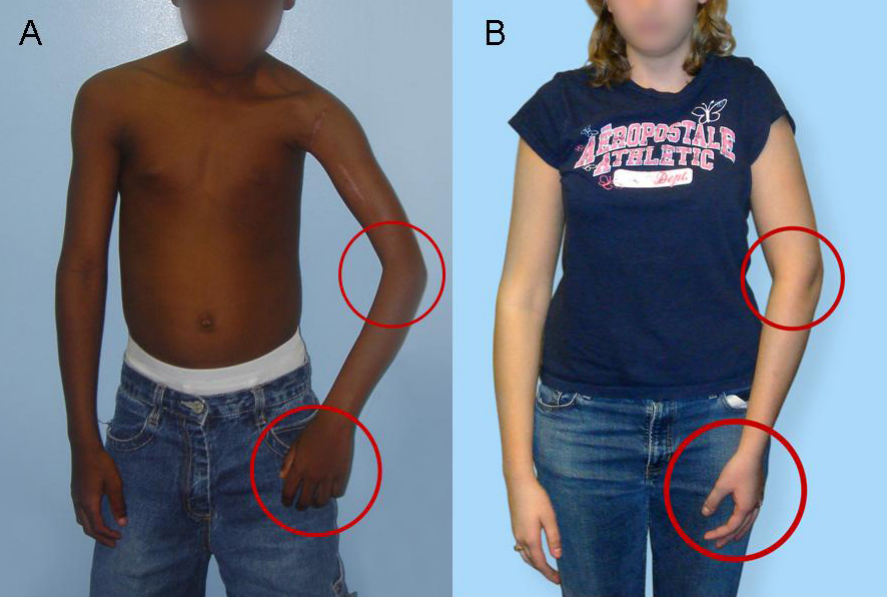
**Neutral position in OBPI patients with medial rotation contracture**. A. A 9-year-old male patient with MRC showing the typical positioning of the elbow crease (towards the body) and with the dorsum of the hand visible anteriorly. B. An 18-year-old female patient with MRC and SD (ARMS variant) (Patient 5). Elbow crease is positioned as the patient in panel A because of the MRC, but the hand appears to be positioned normally because of the coexisting SD. Elbow and hand positioning are highlighted with red circles in both panels

**Figure 2:**
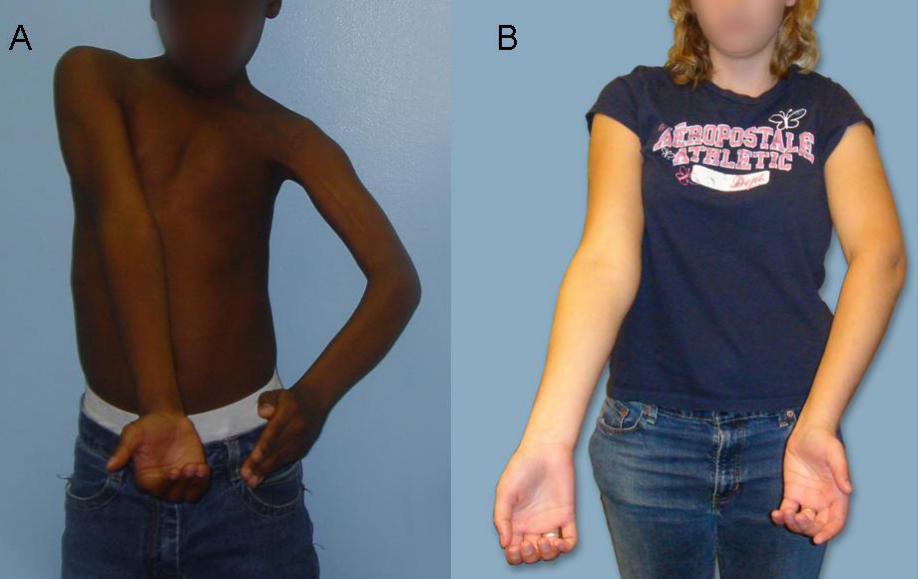
**Apparent supination in OBPI patients with medial rotation contracture**. A. A 9-year-old male patient with MRC showing a lack of supination ability of the left arm due to the medial rotation position of the upper arm. B. An 18-year-old female patient with MRC and SD (ARMS variant) showing apparently normal supination of the left arm (Patient 5). Note the position of the left elbow crease as compared with the right side.

The MRC is a major cause of shoulder deformity in children with OBPI, requiring surgery in more than one third of patients whose injury did not resolve spontaneously [1]. The term SHEAR (Scapular Hypoplasia Elevation And Rotation) deformity has been used to describe the ultimate bony consequence of the muscular fibrosis, and is potentially relevant to the majority of OBPI patients exhibiting MRC [2-4]. The most clearly visible manifestation of the SHEAR deformity is elevation of the scapula[4]. Scapular elevation has also been observed to be further complicated by a forward rotation which occurs along with an abnormal twisting of the clavicle, tilting the entire acromio-clavicular plane forward and causing impingement of the acromion upon the humeral head. [4-6]. Progression of the SHEAR deformity, due largely to unrelieved MRC, may contribute to or further exacerbate the deleterious effect of the MRC on glenohumeral development [7-9]. This is visible as a persistent elbow-bent posture, shortening of the arm and awkward lateral rotation [5,10] (Figure 1A). The act of supination in patients with MRC alone will typically only generate apparent rotation to the neutral position or less, because medial rotation at the level of the shoulder masks the true supination ability (Figure 2A).

Supination contractures of the forearm also cause considerable disability and functional impairment, regardless of the cause or the age of the patient [11-17]. The supinated position of the forearm not only impairs function but also greatly affects appearance, and leads to the "begging hand" posture [16]. The two supinators (biceps and supinator) overpower the pronators (pronator teres and pronator quadratus) creating an imbalance in the forearm during a time of rapid growth. The supination contracture can progressively lead to growth problems in the radial head and even radial head subluxation [16].

Recognition of specific entities and patterns that occur in OBPI patients is important in appropriate management of these deforming and function-limiting deformities. We introduce in this report, a variant of OBPI patients exhibiting Arm Rotated Medially with Supination, the "ARMS" variant of MRC. Further, we discuss the surgical correction of the ARMS variant of MRC with a combination of triangle tilt surgery or humeral osteotomy [2,3] to correct the persistent medial rotation of the arm, and derotational radial osteotomy to correct the forearm supination deformity.

## Methods

### Patient Population

Between February 2005 and June 2006, we identified 14 occurrences of the ARMS variant of MRC among 121 OBPI patients who presented to our clinic for treatment of MRC. Patients were identified by screening for concurrent presence of a fixed supination deformity and medial rotation contracture. The sequence is recognized clinically by the relative positioning of the volar surface of the forearm and the anterior surface of the arm. The forearm volar surface is perpendicular to or over-pronated in relation to the anterior arm surface in unaffected or OBPI patients with MRC alone (Figure 1A). Medial rotation of the arm can be camouflaged by the excessive forearm supination of the supination deformity (Figure 1B). The hand appears to be in the neutral position, but observation of the elbow crease shows that it is turned medially to face the chest wall, and further observation notes the abnormal relationship between the volar forearm and anterior arm surfaces (Figure 1B).

Eight of the 14 patients had sufficient follow-up data for the study (Table 1). This group of patients consisted of 4 males and 4 females ranging in age from 2.2 to 17.9 years (average 7.3 ± 5.1 STD). Five patients had right-handed injuries while three had left-handed injuries. Six of the patients had brachial plexus root injuries confined to C5, C6, and C7 and two had injuries to additional nerve roots. Surgeries performed before this study included modified quad (MQ, N = 7), nerve graft (NG, N = 2), posterior glenohumeral capsulorrhaphy (PGHC, N = 1) and biceps tendon lengthening (BTL, N = 1). This was a retrospective study of patient charts, which exempted it from the need for IRB approval in the United States. Patients were treated ethically in compliance with the Helsinki declaration. Documented informed consent was obtained for all patients.

**Table 1:** Summary of OBPI patients with ARMS variant of the MRC.

						*Pre-operative Values		
Patient	Sex	Injured Side	Current Age (yrs)	Nerve Involvement	Previous Surgeries	PHHA (%)	Glenoid version (°)	SHEAR (%)

1	F	L	5	C5 C6 (C7)	MQ	5	-37	37
2	M	R	7	C5 C6 (C7)	MQ, PGHC	8	-32	49
3	M	R	9	C5 C6 (C7)	none	16	-28	--
4	M	R	12	C5 C6 (C7)	none	11	-33	13
5	F	L	19	C5 C6 (C7)	MQ	48	-8	--
6	M	R	4	C5 C6 C7	MQ	34	-10	19
7	F	L	4	C5 C6 C7 C8	NG, MQ	34	-13	2
8	F	R	10	C5 C6 C7 C8 T1	NG, MQ x 2, BTL	-20	-37	9

(C7) indicates a milder C7 injury, MQ modified quad surgery, NG nerve grafting, BTL biceps tendon lengthening, PGHC posterior glenohumeral capsulorrhaphy,

* Pre- operative values were measured from CT imaging performed before any bony surgeries.

PHHA percent humeral head anterior to the scapular line, SHEAR scapular hypoplasia elevation and rotation represented by scapular elevation.

### Measurements of Glenohumeral joint and SHEAR deformity

Images obtained through CT (computed tomography) or MRI (magnetic resonance imaging) were used to measure glenoid version, posterior subluxation of the humeral head and SHEAR. All measurements were performed by trained scientists independent of the surgeon and principal author.

Glenoid version was measured using the method described by Friedman et al. [18]. A scapular line was drawn connecting the medial margin of the scapula to the middle of the glenoid fossa on transverse images at the mid-glenoid level. The glenoscapular angle, defined as the posteromedial quadrant formed between the scapular line and a line tangential to the glenoid surface closest to the humeral head, was measured and 90° were subtracted to determine glenoid version.

Posterior subluxation of the humeral head was determined using the same scapular line and a perpendicular line traversing the humeral head at its greatest diameter. The distance from the scapular line to the anterior portion of the head and the greatest diameter of the humeral head were measured using the Universal Desktop Ruler (AVPSoft, version 2.8.1110). The ratio of these distances, multiplied by 100 determined the percentage of the humeral head anterior to the scapular line (PHHA) or the extent of posterior subluxation of the humeral head [9].

**Figure 3:**
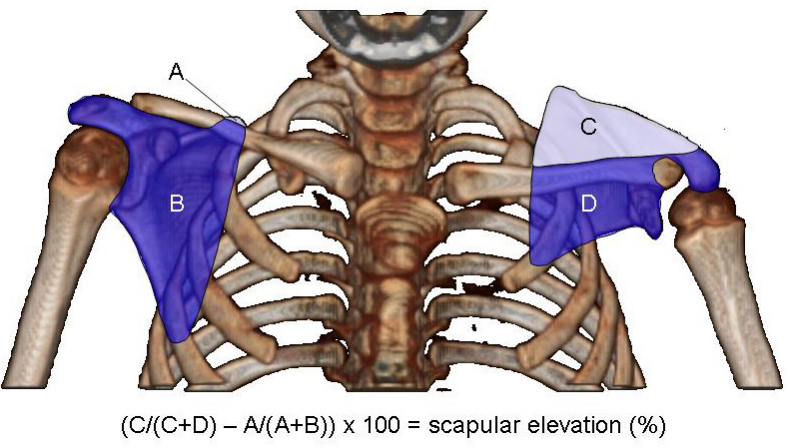
**Measuring scapular elevation to quantitate the extent of the SHEAR deformity**. A 3D-reconstruction of axial bilateral CT images rotated into the anterior view is used to measure scapular elevation. The area of each portion of both scapulas is measured as indicated (areas A-D). The area above the scapula is divided by the total scapular area and corrected for rotational artifacts by subtraction of the unaffected side from the affected side before multiplying by 100 to obtain percent elevation. Shown here is the CT for patient 1 with 37% scapular elevation.

The presence of SHEAR deformity was determined by physical examination and quantitated from 3D-CT images [4]. Elevation of the scapula was estimated clinically by palpation and observation during routine shoulder movements and supination. Scapular elevation was quantified from a bilateral 3D-reconstruction of the CT axial images to determine the severity of the SHEAR deformity [4]. The area of the scapula appearing above the clavicle, measured using Universal Desktop Ruler (AVPsoft, version 2.8.1110), was divided by the total area of the scapula in the anterior view of the 3D-reconstruction. The percent scapular elevation for the unaffected shoulder was subtracted from that of the affected shoulder to correct for rotational artifacts of the anterior projection (Figure 3).

### Evaluation of active arm and shoulder movements

**Figure 4:**
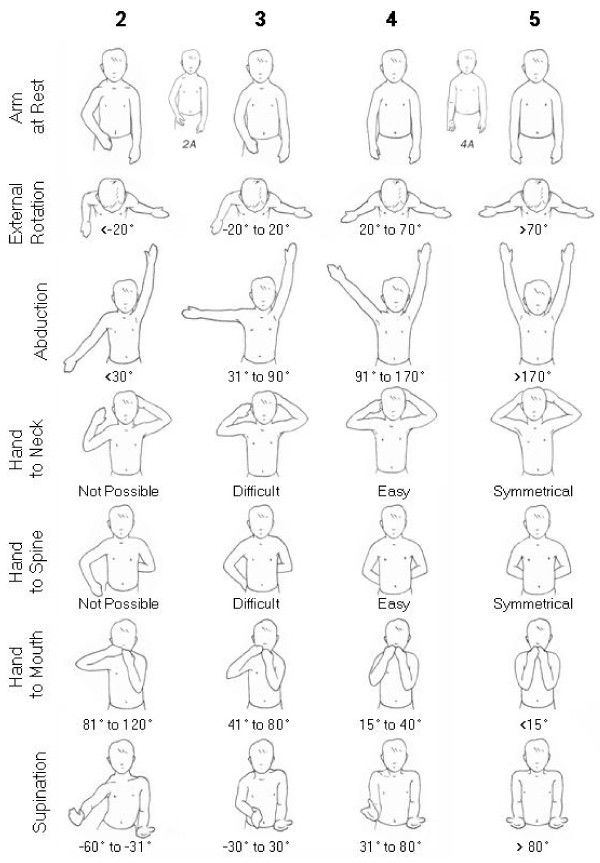
**Modified Mallet scale evaluation of function and arm appearance**. In addition to assessing the classical shoulder functions of the classical Modified Mallet system, supination and the resting position are evaluated. In the resting position, medial rotation at the shoulder is scored on a scale of 1 to 5. Fixed forearm supination is noted in the resting position as indicated by the drawings labeled 2A (first web space visible) and 4A (palm visible). Lateral rotation position can also be noted in the resting position. A total Mallet score is calculated from the scores for abduction, hand to neck, hand to spine, hand to mouth, and lateral rotation, giving a maximum score of 25. Angles are measured from video stills for abduction, hand to mouth and apparent supination and estimated for lateral rotation.

All patients were assessed preoperatively and postoperatively by evaluating video recordings of standardized movements the modified Mallet scale (Figure 4) to index active shoulder movements [19]. In order to more precisely define their functional disability and forearm deformity, the angle of the hand-to-mouth movement (Bugler's sign); and the angle of apparent supination were recorded [2] (0° = neutral position, 90° = full apparent supination, -90° = full apparent pronation) as well as appearance of the upper extremity at rest. Apparent supination is the observed active range of movement that includes both shoulder and forearm contributions to the supination, because the hand position is affected by both rotational components. Although all 7 pictured movements and positions are observed, the overall score is calculated for only five as in the modified Mallet scale [19]. All evaluations were conducted by trained scientists independent of the surgeon and lead author.

### Statistical Analyses

Paired Student's t-tests were conducted using Microsoft Excel 2003 (Redmond, WA) to determine if differences between preoperative and postoperative Mallet scores for each function were statistically significant. The p values were two-tailed and considered significant if less than or equal to 0.05.

### Surgical correction

The MRC was corrected as the first stage in management of the ARMS variant, with SD correction performed several months later to complete the surgical sequence. In addressing the MRC, the lead author and the surgeon (RKN) of this report performed a novel osseous procedure, named the triangle tilt. This procedure releases and tilts the acromio-clavicular plane back to neutral from an abnormally forward-tilted position, thus relieving impingement of the acromio-clavicular triangle upon the humeral head [2,3]. The triangle tilt surgery consisted of four major components. Firstly, osteotomy separated the clavicle at the junction of the middle and distal thirds. Secondly, osteotomy of the acromion process at its junction with the spine of the scapula was performed. Thirdly, osteotomy of the superomedial angle of the scapula was performed to relieve soft tissue impingement against the abnormally rotated superomedial angle of the scapula. Finally, the extremity was splinted in lateral rotation and full forearm supination (90°). Splinting was maintained for 6 weeks after which time the splint was worn only at night for an additional 3 months. Minor elements of the procedure included bone grafting of the acromion process osteotomy site, and semi-rigid fixation of the clavicular osteotomy segments to prevent nonunion. One patient in the study was too old to undergo triangle tilt surgery and underwent humeral osteotomy as correction of the medial rotation contracture.

The SD was addressed as a second stage surgical correction with derotational osteotomy of the radius and intramedullary pinning. A small percutaneous incision was made directly over the distal aspect of the radius, and blunt dissection with a hemostat was taken down to the metaphysis of the distal radius. A medium-sized Steinmann pin was then utilized as an L and a cortical window was made in the metaphysis. A 2 mm Steinmann pin was then bent 20 degrees of its tip, advanced down this cortical window until midshaft of the radius. The pin advancement was stopped and lines were drawn on the skin marking the advancement point. A 2 to 3 cm longitudinal incision directly over the radial border of the forearm was then made. The radius was exposed subperiosteally. A small oscillating saw was then utilized to make a transverse osteotomy which was completed with an osteotome. The Steinmann pin was then advanced past the osteotomy site into the proximal radius. It was bent outside the skin and buried. Forearm rotation was tested for acceptability in each case. The wounds were irrigated and closed. The patient recovered in a semi-rigid fiberglass cast with the elbow at 90 degrees flexion and the forearm at 10–20 degrees of pronation as functional for the patient.

## Results

**Figure 5:**
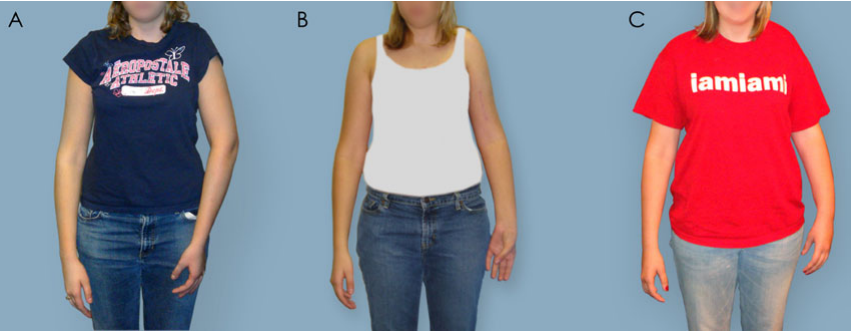
**Arm at resting position**. The patient was asked to stand with her arms at resting position. A: Patient 5 prior to any surgery. B: Patient 5 after shoulder correction surgery. C: Patient 5 after forearm osteotomy surgery. Changes in the relative positioning of the volar surface of the forearm to the anterior surface of the arm are visible in the affected left arm.

**Figure 6:**
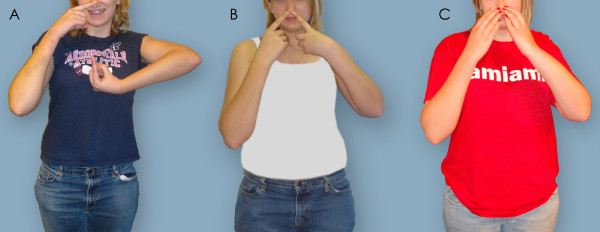
**Hand to nose**. The patient was asked to touch her nose with both her hands. A: Patient 5 before any surgery. B: Patient 5 after shoulder correction surgery. C: Patient 5 after forearm osteotomy surgery. Changes in functional position of the left arm and hand are visible.

**Figure 7:**
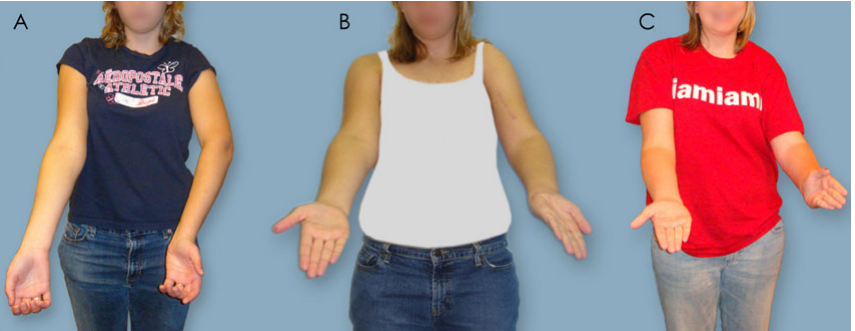
**Supination**. The patient was asked to place her arms in front of her with her palms facing the ceiling. A: Patient 5 before any surgery. B: Patient 5 after shoulder correction surgery. C: Patient 5 after forearm osteotomy surgery. Changes in the positioning of the elbow crease and palm are visible in the affected left arm.

Figures 5, 6, 7 depict preoperative versus postoperative changes for the arm-at-rest position, hand-to-mouth movement, and apparent supination in a representative OBPI ARMS variant patient who underwent the recommended sequence of shoulder reconstruction followed by forearm osteotomy (Patient 5, Table 2). Each figure is formatted in the following manner: 1. panel A shows a picture of the patient before any bony surgeries; 2. panel B show a picture of the patient after the shoulder reconstruction; and 3. panel C shows a picture of the patient after the forearm osteotomy.

**Table 2:** Mallet scores for the eight patients.

Pre-operative Function									Surgery Dates			Post-operative Function								
				
Patient Number	Abduction	Hand to Neck	Hand to Spine	Hand to Mouth	Hand to Mouth ∢ (°)	Lateral Rotation	Total Mallet	Supination ∢ (°)	Triangle Tilt	Humeral Osteotomy	Forearm Osteotomy	Date	Abduction	Hand to Neck	Hand to Spine	Hand to Mouth	Hand to Mouth ∢ (°)	Lateral Rotation	Total Mallet	Apparent Supination ∢ (°)
1	4	2	3	2	90	2	13	-45	04/06			02/07	4	3	2	4	30	4	17	50^†^
2	5	2	2	2	110	4	15	35	05/05		01/06	08/07	5	4	4	4	20	3	20	20
3	4	2	2	2	90	3	13	30	11/05		05/06	07/07	4	4	4	5	0	4	21	60
4	4	2	2	2	90	4	14	45	11/05 06/06			04/07	5	4	4	4	20	4	21	60^†^
5	3	3	2	1	150	2	11	-45		07/06	11/06	05/07	4	4	3	4	20	4	19	50
6	4	2	2	3	80	4	15	50	05/05		10/05	04/07	4	4	4	4	25	4	18	40
7	4	3	2	2	100	2	13	-45	11/05			04/07	4	4	4	4	40	5	19	90^†^
8	5	3	3	1	150	2	14	-45	02/05	11/06	11/05	06/07	5	3	1	1	120	3	14	0

	4.1	2.4	2.3	1.9	108	2.9	13.5	-2.5	pre-op mean			post-op mean	4.4	3.8	3.3	3.8	34.4	3.9	18.6	34^†^
												p-value**	0.2	0.001	0.4	0.001	<0.001	0.07	0.001	0.2^†^

*For date, the first two numbers indicate the month and the last two numbers indicate the year. Pre-operative function was determined from videos taken the day before the earliest surgery.

** Two-tailed p-value from paired Student's t-test

† These patients will have a reduction in apparent supination angle after forearm osteotomy. The mean and p-value represent only those 5 patients with completed reconstructions.

Figure 5 shows preoperative and postoperative changes in the arm-at-rest position. In Figure 5A, the hand position of the injured arm appeared similar to the contralateral arm. In particular the forearm of the injured arm appeared to be in a neutral position which was in contrast to the apparently pronated position of the forearm seen in many OBPI patients with MRC. Unlike the contralateral arm, the elbow crease and the dorsum of the hand were not clearly visible when the injured arm was at rest. The picture in Figure 5B shows the effects of the humeral osteotomy on the position of the injured arm at rest. The elbow crease was visible on both the injured and contralateral arms; however, unlike the contralateral arm, the volar surface of the forearm and hand were clearly visible in the injured arm. In Figure 5C, both forearm and hand of the injured arm were in a neutral position after forearm osteotomy.

Figure 6 shows preoperative and postoperative changes in the hand-to-mouth movement. The patient could not achieve this movement prior to the surgeries (Figure 6A). After humeral osteotomy, the patient could touch her nose with her injured arm at a 40° angle (Figure 6B) which further decreased to 20° after forearm osteotomy (Figure 6C). Figure 7 shows preoperative and postoperative changes in apparent supination. In Figure 7A, the positions of both hands were similar; but no elbow crease was present in the injured arm. In Figure 7B, the elbow crease was present while the injured arm was supinated, but the angle of the hand was greater than 90°. In Figure 7C, both arms and hands were in similar positions.

### Mallet grading

The preoperative and postoperative Mallet scores for the OBPI ARMS variant patients are presented in Table 2. The preoperative Mallet evaluations were conducted before the first bony surgery (i.e., triangle tilt, humeral osteotomy, or forearm osteotomy) while the postoperative Mallet evaluations were conducted after the final bony surgery and represented the most recent values for these patients. The number of months between the two Mallet evaluations ranged from 10 to 28 months with an average of 18.7 months. During this time period, seven out of the eight patients underwent triangle tilt surgeries. Four of these triangle tilt patients later had forearm osteotomies (one of the patients had also undergone a humeral osteotomy). One patient did not have triangle tilt but underwent humeral osteotomy due to her age, followed by a forearm osteotomy.

The mean Mallet score significantly improved from a preoperative mean value of 13.4 points (range 11 to 15) to a postoperative mean value of 18.6 points (range 14 to 21) (p = 0.001, Table 2). Abduction did not significantly improve (p = 0.170), because the majority of ARMS variant patients had previously undergone modified quad surgery, which has been shown to improve global abduction in OBPI patients [20]. Hand to neck movements significantly improved (p = 0.001, Table 2) from a preoperative mean of 2.3 (range 2 to 3) to a postoperative mean of 3.8 (range 3 to 4). Hand to mouth movements occurred in an improved functional plane: trumpet sign angle significantly decreased from a preoperative mean of 107.5° (range 80° to 150°) to a postoperative mean value of 36.9° (range 0° to 120°; p < 0.0001, Figure 5). Mallet scores for lateral rotation also increased from a preoperative mean of 2.9 (range 2 to 4) to a postoperative mean of 3.9 (range 3 to 5); however, the improvement in scores was not statistically significant (p = 0.068). The angles for apparent supination of the forearm increased from a preoperative mean of -5.0° (range -45° to 50°) to a postoperative mean of 34.0° (range 0° to 60°) (p = 0.222, Figure 7) for the five patients who completed both reconstructive surgeries. Forearm osteotomy improved the passive range of motion, but not active range of motion of the forearm, and improved the functional positioning of the palm. The radial osteotomy to correct a supination deformity rotated the forearm into a more pronated position. This was seen in the at rest position and during supination, as recorded in the apparent supination angles.

## Discussion

Supination is one of the most misunderstood concepts in arm movement, especially in the context of brachial plexus injury. Lack of supination results in abnormal postures of the forearm and hand, the "waiter's tip" position, and excessive supination is sometimes known as the "begging hand" position. All patients in our study did have supination deformity of the forearm, but this was not immediately obvious on initial physical examination due to the excessive medial rotation of the arm in conjunction with coexisting supination deformity. The simultaneous presence of medial rotation deformity of the arm and a supination deformity of the forearm will give a neutral-appearing hand position (Figures 1 and 2). Functionally, this is important because it substantially impairs utility of the extremity (Figures 6A and 7A). We address the simultaneous occurrence of the MRC and the SD in a group of patients with a history of OBPI. The clinical features of the entity offer an apt description of the overall deformity: Arm Rotated Medially with Supination (ARMS). The ARMS entity is actually best thought of as a variant of the MRC, hence the appropriate term is "ARMS variant of MRC".

Most of the ARMS variant patients in our study group had C5–7 injuries (75%), whereas 25% of them suffered additional C8 and T1 root injury. In all cases, supination function of the biceps recovered more completely or earlier than pronation function. Even when innervation of pronators is recovered, the earlier recovery of powerful biceps can be difficult to overcome. Sibinski and colleagues observed that even though the initial injury causes greater weakness to supinators than to pronators, the long term outcome was limited pronation in 86% of their patients with evidence for a mild supination contracture in 34% of all patients [21]. It is possible for this to occur when the injury to C7 is transient and the biceps (C5–C6) recover power before the pronator teres (C6–C7), leaving it unable to overcome the supination force of the biceps. Although the pronator quadratus (C8-T1) may also be contributing some pronation force in the scenario where the lower roots are unaffected, it is apparently not great enough to prevent the imbalance from developing into a fixed supination deformity. A study by Gordon and colleagues of EMG signal during supination and pronation [22] suggests that the pronator teres muscle is the primary agonist during resisted pronation (as in these patients: the early recovery of biceps supination power). The net result is development of supination deformities due to relative pronation weakness with excessive supinator and biceps muscle activity [23,24].

The management of the ARMS variant of MRC requires attention to both medial rotation contracture as well as a supination deformity. Traditional surgical treatment for MRC has included humeral osteotomy as well as L'Episcopo-type tendon transfers [25]. Neither one addresses or corrects the shoulder subluxation, and therefore does not correct the main cause of disability in these patients. We have addressed the MRC by using the triangle tilt procedure. This method was followed since the SHEAR deformity is the underlying pathophysiology behind the fixed medial rotation contracture. We have previously reported the successful use of the triangle tilt procedure to correct the MRC in 44 OBPI patients [2], and continue to see improved shoulder function in this set of ARMS variant patients. One patient's MRC was treated with a humeral osteotomy, because of low SHEAR and age-related increased ossification.

Correction of supination deformity is obtained either by biceps rerouting, or rotational osteotomy of the radius [16,26-28] and/or ulna [23]. The biceps rerouting has been performed in children with good passive forearm pronation (indicating the lack of contracture of the interosseous membrane), strong biceps, and no radial head dislocation. The osteotomy procedure has been carried out if there is contracture of the interosseous membrane weak biceps, or dislocation of the radial head [16,23,26,27]. Birch et al.,1998 [5], have also reported joint release and tendon transfer procedures to improve hand and elbow functions. More recently, Ozkan et al., 2004 [29] proposed a brachioradialis muscle re-routing as an alternative to the biceps re-routing.

The fixed supination deformity was addressed in five of our patients with derotational radial osteotomy resulting in correction of forearm position and some improvement in passive range of motion. By using the triangle tilt surgery in conjunction with the later radial osteotomy the arm posture in the resting position was visibly improved (Figure 5). Although the forearm is rotated towards pronation during the forearm osteotomy, the net outcome from both shoulder and forearm reconstruction is an insignificant (p = 0.2) gain in maximum apparent supination angle (from 5° to 34°). The two surgeries have opposite effects on apparent supination angle, but Mallet scores significantly (p < 0.05) show that these surgeries together improved function. These children and their parents are pleased with the appearance of the arm, forearm and hand and also with their increased ability to carry out their activities of daily living.

## Conclusion

In this report, we describe a subset of OBPI patients with a medial rotation contracture of the arm with supination of the forearm, Arm Rotated Medially with Supination (ARMS) variant of MRC. The OBPI ARMS variant patients will require separate surgical procedures to correct each deformity. Improvement in function was statistically significant.

Based on our experience, we recommend that ARMS variant patients undergo staged surgical correction of the medial rotation contracture followed by derotational radial osteotomy to correct the supination of the forearm. In our study, we found that the final effects of medial rotation contracture correction by triangle tilt not only returned the arm and shoulder to a neutral position but also revealed the true extent of fixed supination within the forearm (Figure 5B). In addition, it has been shown that correction of the medial rotation of the affected arm in OBPI patients enhanced lateral rotation [2,30,31]. Therefore, we believe that the medial rotation of the arm should be alleviated before the forearm supination deformity can be accurately assessed in OBPI ARMS variant patients.

## Competing interests

The authors declare that they have no competing interests.

## Authors' contributions

RKN conceived the study, participated in the design of the study and drafted and revised the manuscript. CS participated in the design of the study, gathered data, helped to draft and revised the manuscript. SEM participated in the design of the study and revised the manuscript. MB gathered data, and helped draft the manuscript. MJW participated in the design of the study and helped draft the manuscript. All authors have read and approved the final manuscript.

## Pre-publication history

The pre-publication history for this paper can be accessed here:


